# How do I keep myself safe? Patient perspectives on including reason for use information on prescriptions and medication labels: a qualitative thematic analysis

**DOI:** 10.1186/s40545-020-00268-6

**Published:** 2020-10-07

**Authors:** Colin Whaley, Ashley Bancsi, Joanne Man-Wai Ho, Catherine M. Burns, Kelly Grindrod

**Affiliations:** 1 School of Pharmacy, University of Waterloo, 200 University Ave West, Waterloo, ON N2L 3G1 Canada; 2 Division of Geriatric Medicine, Department of Medicine, McMaster University, 1280 Main Street West, Hamilton, ON L8S 4K1 Canada; 3 Division of Clinical Pharmacology and Toxicology, Department of Medicine, McMaster University, 1280 Main Street West, Hamilton, ON L8S 4K1 Canada; 4 Schlegel-UW Research Institute for Aging, 250 Laurelwood Drive, Waterloo, ON Canada; 5 GeriMedRisk, 250 Laurelwood Drive, Waterloo, ON Canada; 6 Department of Systems Design Engineering, Faculty of Engineering, University of Waterloo, 200 University Ave West, Waterloo, ON N2L 3G1 Canada

**Keywords:** Drug safety, Medicine policy, Pharmacy, Primary care

## Abstract

**Abstract:**

**Background:**

Medications are crucial for maintaining patient wellness and improving health in modern medicine, but their use comes with risks. Helping patients to understand why they are taking medications is important for patient-centered care and facilitates patient adherence to prescribed medications. One strategy involves enhancing communication between patients, physicians, and pharmacists through the sharing of reason for use (RFU) information or the indication for medications.

**Methods:**

Semi-structured interviews were conducted with 20 patients in Ontario, Canada, to gain perspectives on how patients currently store their medication information and benefits and disadvantages of adding RFU to prescriptions and medication labels. An interview guide was used by the two interviewers, and the interviews were recorded, transcribed, and thematically coded.

**Results:**

The analysis yielded three main themes: patient decision making with RFU, RFU in modern, patient-centered care, and logistical aspects of communicating RFU. The patients that were interviewed expressed the value of having RFU when deciding if a medication was effective or to stop taking the medication. Patients felt comfortable with RFU being added to prescriptions and acknowledged the value in adding RFU to medication labels, helping patients and others identify and distinguish medications. Patients generally expressed interest in having RFU written in lay language and identified strengths and weaknesses of having access to RFU via a website or app.

**Conclusions:**

Patients rated the importance of knowing RFU very highly, identified the value in sharing RFU with pharmacists on prescriptions, and in having RFU on medication labels. These results can be used to inform policy on the addition of RFU on prescriptions and medication labels and support improved communication between patients, pharmacists, and physicians about RFU.

## Introduction

**Fig. 1:**
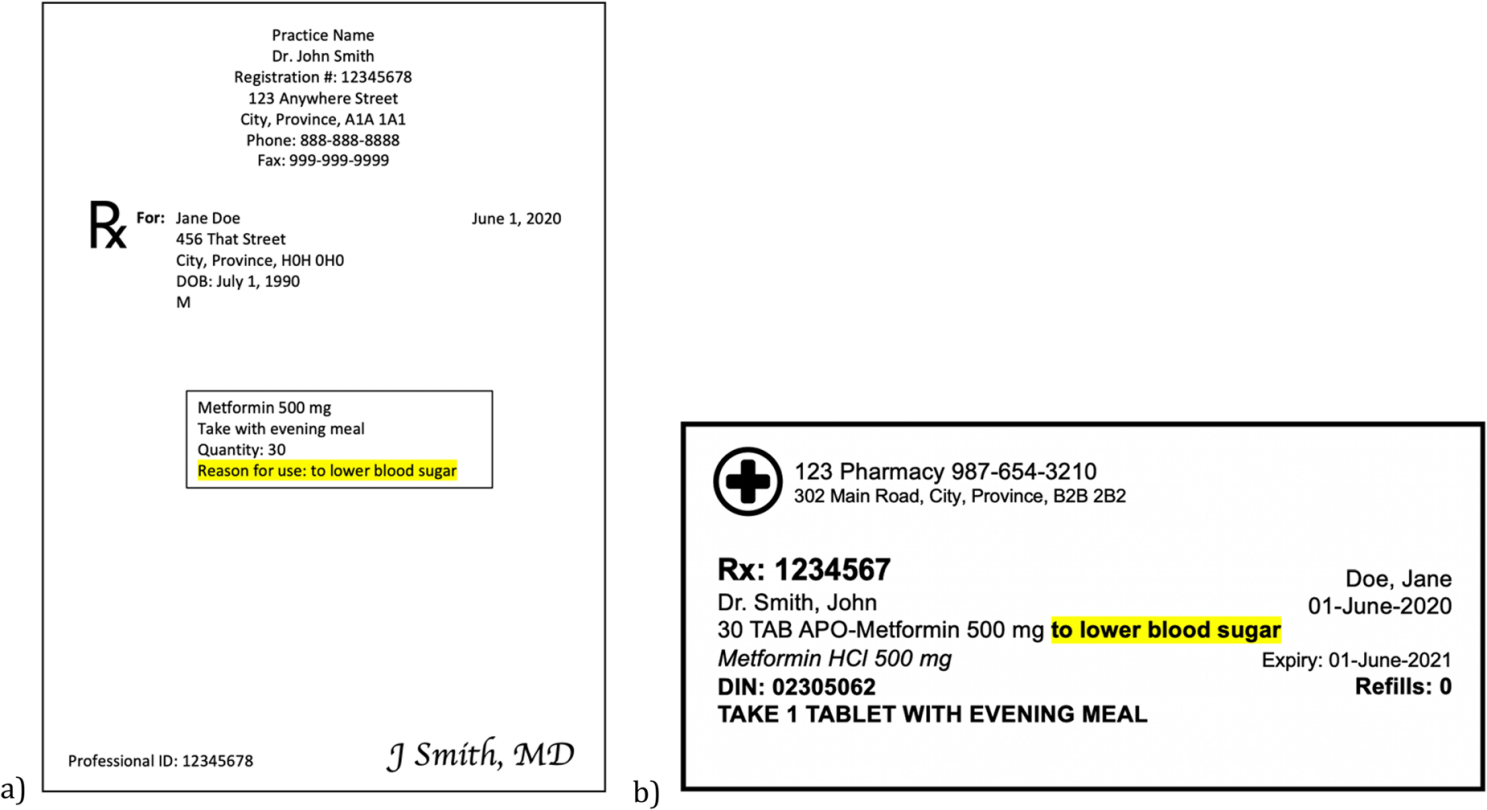
Mock-ups of the addition of RFU on **a** prescriptions and **b** medication labels. The highlighted area indicates RFU. **a** Based on the prescriptions generated by PS Suite, TELUS Health [3]. **b** Based on the medication labels produced by Kroll™, TELUS Health, with the location of RFU from Hussein, 2018 [4, 5]

Medications are fundamental for the maintenance of good health and the treatment of disease in modern medicine. In Canada between 2007 and 2011, 41% of people living in the community between the ages of 6 and 79 took at least one medication, and around 30% of 65- to 79-year-old people experience polypharmacy, taking 5 or more distinct medications per day [1]. With adverse drug events causing more than 27,000 hospitalizations in Canada between 2010 and 2011, the safe prescribing and use of medications, as well as improving clinician-clinician and clinician-patient communication around medications, represent key areas for health systems to facilitate safe medication use [2]. One potential strategy is the addition of medication indications, also known as the reason for use (RFU), onto prescriptions, and medication labels. Example designs of prescriptions and medication labels with RFU included can be seen in Fig. 1 [3–5].

Patients are increasingly being asked to manage many aspects of their care, including communicating health information between healthcare providers and maintaining records of their own health [6, 7]. Numerous studies have indicated that patients are sometimes unaware or incorrect about why they are taking a particular medication [8, 9]. Adding RFU to medication labels would allow patients to always have RFU along with their medications and could improve adherence to prescribed medication treatments [6].

Prescribers have identified barriers to the addition of RFU on medication labels, including the time required to add indications when writing a prescription, uncertainty of how to format the information, and being unsure of the value of adding RFU to prescriptions [10]. However, they acknowledged potential time savings in including RFU by pre-empting concerns from pharmacists [10]. From a pharmacy perspective, pharmacists have indicated that they would be able to more effectively carry out their clinical duties if provided RFU on a prescription. Providing pharmacists with access to RFU has been shown to nearly double the number of prescribing errors they detect [11].

A systematic review identified that medication labels facilitate communication and comprehension about medications by patients and that medication use errors may be caused by poor medication labels [12]. Additionally, medication labels designed to be more patient-friendly have been shown to improve adherence in patients with low literacy, as well as for medications, that need to be taken two or more times per day [13]. Through these studies, however, a clear understanding of how patients themselves may use RFU was not determined. Thus, the objective of this paper is to describe patients’ perspectives on the usefulness of adding RFU information to prescriptions and prescription labels and how patients may use RFU to make decisions about their medications.

## Methods

Semi-structured interviews were conducted with 20 community members who have used at least one medication in the last 30 days. We decided to conduct 20 interviews on the basis of feasibility to define a clear end-point for conducting interviews, which is in line with existing literature recommendations [14, 15]. We did not aim to capture a maximum-variation sample. Patients were recruited via flyers posted in public places, including the local university, doctor’s offices, and pharmacies. Patients were also recruited using an institutional database of older adults who indicated an interest in being contacted about research studies. Two individuals who inquired about participation were ineligible due to not meeting the medication-use requirement. All participants were from Southwestern Ontario and were interviewed at a time and place of their choosing. Semi-structured interviews allowed for flexibility on the part of the researchers to follow-up on statements and themes mentioned by participants [16]. This study was approved by a research ethics committee. Participants were asked questions about how they organize information about their medications, their comfort with having RFU communicated to pharmacies, and their thoughts on having medications’ RFU on medication labels. A $25 CAD honorarium was given to participants in thanks for their time. Information on participants’ demographics and the number of medications were collected.

### Data collection

Interviews were conducted by two pharmacy researchers and one system design engineering researcher, using the interview guide in Appendix A. This interview guide was developed by the pharmacy and system design engineering teams, with input from patient partners. The interviews were recorded and transcribed.

### Data analysis

NVivo for Mac was used to store and analyze the interviews [17]. Thematic analysis was used for the analysis, due to its flexibility in capturing both major themes and deviant cases across the interviews. Pharmacy researchers C.W. and K.G. analyzed the first five interviews and developed a working codebook. Differences in codes developed, as well as the codes assigned to the interviews, were resolved. C.W. and K.G. discussed codes and additions to the codebook every subsequent 5 interviews analyzed. Codes were then organized into broader themes separately by C.W. and K.G. C.W. selected the final themes and codes in collaboration with K.G., and a framework matrix was generated using NVivo 11 for Windows. The matrix was used to select quotes for inclusion in the themes, where quotes expressing the majority’s opinion as well as divergent cases were included. C.W. wrote memos throughout the interview analysis process to make note of key quotes and themes as they developed. For the 20 interviews analyzed, inductive thematic saturation is claimed on the basis of no new codes emerging [18]. The Standard for Reporting Qualitative Research (SRQR) were followed during the preparation of this manuscript [19].

Participants were also asked to participate in an activity to redesign a prescription label, which informed the sample medication label in Fig. 1 [5]. Those results, as well as data from some of the interviews, are published elsewhere [5, 20].

## Results

The 20 patients interviewed were primarily adults and older adults and took a median of 4.7 medications (IQR = 3–6). Ten males and ten females were interviewed. Additional details can be found in Table 1. Patients generally acknowledged the value of providing pharmacists with RFU and having RFU on prescription labels, while acknowledging that including RFU on a prescription label may pose a privacy risk.

**Table 1:** The 20 patients interviewed

Demographic information on participants	
Gender	
10 males	
10 females	
Age	
Young adult (15 to 24 years): 1	
Adult (25 to 59 years): 7	
Older adult (60+): 12	
Number of regular/long-term medications number of medications	
Minimum: 0	
Average: 4.7	
Maximum: 10	
Mode: 3	
IQR = 3–6	

### Theme 1: patient decision making with RFU

Patients framed their understanding of RFU as the reason a medication was prescribed (i.e., to decrease my blood pressure), as opposed to what it was treating (i.e., hypertension). The notion of prescribers explaining the rationale for a medication’s use was reflected in patients valuing RFU in helping them make decisions regarding the use of the medication. For example, patients might use RFU to know if they are using medications correctly.

I would say that the biggest implication is not using it correctly. I know with the cream and with just some other things when I was trying migraine corrections, when I don't know why and I don't exactly know the "how" and the reason why it's important to take it at the same or anything, I would take it incorrectly and then it wouldn't have the desired effect. [Patient 006]

As well, others used RFU to make decisions regarding the continued use of a medication:

Well, you would know how important it was. You wouldn't want to skip doses if it was something very vital. [Patient 012]

And to understand whether a medication was working:

If your symptoms get worse, or your overall condition changes, you can kind of use your discretion on whether you should continue using it or not instead of having to go to the doctor each time. [Patient 005]

In all of these cases, providing RFU in an easily accessible manner would allow the patient to make more informed decisions about their care and help differentiate medications if RFU was included on a label. One patient described how they currently draw symbols on their medication labels to tell them apart:

… having a label that would say use this for this or this for that, would make sense to me to have that on it. I truthfully, when I'm getting prescriptions, I put them in my medicine cabinet and I will on my Luvox, my antidepressant, I put a happy face on it. Or on the Celebrex, I just mark on it bones. [Patient 020]

Additionally, having RFU on medication labels would also be helpful for instances where there is a change in the brand of medication dispensed. One patient described their frustration with medication information receipt as a way to organize RFU as follows:

…over the years, the [drug store] changed, maybe to a different brand, and the name changes. So if I look at my original [medication slip] I don't know what the new ones are. [Patient 015]

Finally, patients appreciated that having RFU along with a medication name would allow them to learn additional information about their medication online:

…when [my family doctor] gives me prescriptions he explains to me at the time why he is giving me something and what it should do for me. I then go online and look out to see what the side effects might be. [Patient 020]

### Theme 2: RFU in modern, patient-centered care

Patients were asked to reflect on times they did not know the RFU for their medications. Some older participants recounted anecdotes like the following:

When I was younger, the doctors just prescribed stuff and you accepted what they said without question. That was the mentality of the time, the doctor was this all-powerful all-knowing figure, and you were just the consumer of his services and you were expected to blindly accept what the doctor told you. [Patient 017]

In accordance with practices relating to patient-centered care, this mentality has shifted to acknowledging patients as partners in care. One participant, a retired nurse, succinctly described the value of RFU to patients:

If [adding RFU] were to be implemented across the board, I think it would… give people the opportunity to ask the “whys” and it would give them opportunities to find out more about the medications they're on. [Patient 007]

When asked to rate the importance of receiving RFU from their prescriber out of five, 19 of the twenty patients rated it at least a five out of five, with a number of patients exceeding the upper bound provided. All of the patients interviewed expressed a desire to have a deeper level of understanding of their medications, for example, understanding their physicians’ decision-making process:

Well, I want to know that my doctor understands why he's prescribing this drug. Whether he's prescribing it for its mainly intended use, or whether it's for an off-label use… I think patients these days want to be more proactive in their own healthcare. [Patient 017]

Patients were on-board with RFU being shared with pharmacists, and many reported receiving varying levels of counseling from their pharmacist, ranging from quick check-ins for repeat prescriptions, to yearly comprehensive medication reviews.

I have no problem with [reason for use information being shared with my pharmacist on every prescription] because, to me, [both pharmacists and prescribers] are providing a professional service, and the more they're talking, the better… for everybody. [Patient 014]

Additionally, the majority of the patients reported that at least one family member was aware of why they were taking their medications.

Well, I would think your husband or wife should know, and if your children are around, if they're adults they should know. But I don't think the whole world needs to know. [Patient 013]

All patients were able to note some benefits of adding RFU to medication labels. These included helping patients with polypharmacy manage their medications [Patient 007, 014], providing information to others in emergency situations [Patient 006, 010, 011, 015], and distinguishing medications from each other [Patient 001, 016]. Additionally, patients identified situations where other people are responsible for medication administration [Patient 013, 014, 017] and for older adults who need support [Patient 014, 019] as other times when having RFU on medications would be particularly valuable. Regarding emergencies, one of the participants shared the following:

Well, I think in an emergency situation, it would be good for somebody if they saw [the RFU on my medication’s label]. Especially if… they found it in my purse, then they would know, okay yeah, she's been taking this for X number of years. [Patient 011]

Inversely, patients also readily identified a number of potential disadvantages to adding RFU medication labels, including other people potentially seeing the RFU [Patient 003]. This included including stigma surrounding sexually transmitted infections [STIs] or psychiatric illnesses [Patient 007] illnesses one does not wish to disclose to others (i.e., family) [Patient 013], and the potential for teenagers to bully each other as a result of RFU information [Patient 019].

### Theme 3: logistical aspects of communicating RFU

When asked, patients readily provided a number of methods they use to organize information related to their medications (Table 2). To organize their medications, most patients reported either keeping a list or keeping the indications in their memory.

**Table 2:** Patient reported methods of organizing medication information

Ways of organizing medication-related information	
A notebook with all medical-related information	
A list of medications (mentioned by a majority of patients)	
One patient mentioned keeping it in their first-aid box	
Another, in triplicate, one with them, two at home	
Keeping the medication bottles in one place	
Remembering it (mentioned by a smaller number of patients)	

When asked about the prospect of adding RFU to a medication label, the majority of participants agreed with the idea of adding RFU to medication labels. A number of patients specifically noted the value of adding the RFU close to where the directions are:

I think it should be right under where the instructions are of how to take it. The average person doesn't want to know exactly the name of the prescription, they may not understand what the name of the drug is. [Patient 017]

Patients were asked how they wanted RFU to be presented in terms of the language used. Patients preferred that RFU be presented in a way that respects their knowledge and understanding of medications. For some patients, this meant lay language:

Just tell me straight up… Just not technical stuff, not technical. [Patient 010]

And for others, more medical language:

…I'm a public health major and I told [the doctors I am comfortable with medical language], so they were able to say, what is calcification and hypertension, and even words like ... embolization and embolization processes, 'cause it's one of the surgeries I could opt for. I know what that is, so they were very comfortable just using textbook words with me. [Patient 001]

Patients stated they expected that the RFU would be between one word to one sentence in length. “As brief as it could possibly be,” as Patient 018 put it.

When presented with the option of accessing information about their medications using a web-based system (e.g., website, mobile app), patients had mixed feedback. One patient discussed their perception of how frustrating managing login information for different systems could be:

…[if one system] connect[s] to hospital, I might use a [Medical Record Number], I might use a hospital ID, and it's usually not the same for each hospital. For me, with so many specialists at different hospitals, I could just imagine it being a mess. Maybe I use the wrong ID for the wrong hospital, then and I get frustrated. [Patient 001]

Similarly, a different patient was concerned with different pharmacies using proprietary systems that do not allow information to be transferred between pharmacies:

The concern I have with an app is that because I have to move around occasionally as I'm not always convinced that the pharmacy will be connected to the right app. Then I'd have to have multiple apps so I find that a bit more burdensome…. unless [there] was a third-party system that all pharmacies were forced to be on I guess. [Patient 002]

Likewise, privacy concerns were raised by some participants who were afraid that their protected health information (PHI) would be accessed by unauthorized users:

…because it's too easy to access by people who want to pry for nefarious reasons. [Patient 008]

However, the idea of using a web-based system to access information about medications was generally regarded as an attractive option, exemplified by this participant’s viewpoint as an older adult:

I think as the boomers age, more and more of that's going to be possible because we're becoming more and more tech savvy. And if I don't understand, I can ask my nine-year-old grandson who will tell me. [Patient 007]

## Discussion

In the interviews, patients considered the RFU to be the rationale behind prescribing the drug. This information is useful for patients to help make decisions around medication use, such as knowing which medications would treat which symptoms or determining when a medication could be stopped. Patients also felt that the RFU could help them to organize medication-related information, work with others such as family members to manage their medications, or communicate with emergency medical personnel. In contrast, in prior research, pharmacists saw RFU as a tool to assess medication safety, and physicians saw it as a tool to support interprofessional communication (i.e., to decrease the number of clarifying calls from the pharmacy), and to provide transparency around why a drug is prescribed [10]. Of note, no singular terminology or definition about what a clinical indication is or what information it needs to carry exists [21]. Indications listed on medication bottles should facilitate use by patients for decision making, as well as being of utility for clinicians to facilitate information transfer. However, like the pharmacists and physicians interviewed previously, the patients interviewed expressed concerns about privacy if someone saw RFU on a prescription label [10].

Many of the older participants noted that how their physician communicates with them has changed over time, representing a shift to patient-centered care. Communicating RFU represents a change in how information communicates previously [22]. However, given the gap between clinicians’ and patients’ understanding of what RFU is, clinicians will need to work alongside patients to ensure that when a medication is prescribed, information pertaining to both the symptoms and diagnosis is communicated clearly to patients. Increasing the accessibility of RFU is complementary to current patient medication safety initiatives, including Canadian Patient Safety Institute’s *#ConquerSilence* [23, 24] and *Five Questions to Ask about your Medications* [25] campaigns to increase transparency and information sharing about medications. Additionally, it directly aligns with a number of guidelines from the Institute for Safe Medication Practices, including their *Guidelines for Standard Order Sets,* [26] and published strategies to mitigate errors associated with look- and sound-alike medications [27, 28].

Patient participants indicated a desire to have RFU communicated in the language they could easily understand, which was often lay language. This finding aligns with existing work in this area and reinforces the importance of RFU as a tool to support patients in taking their medications [6]. Additionally, all participants were able to determine a situation in which having RFU written on medication labels would be valuable. Many of these were situations were where the RFU would be interpreted by a third party, and in a number of the cases, a person without medical training. Given this context, writing RFU in lay language on medication labels would provide the most utility in the broadest number of situations, allowing patients, family members, and others to understand what a particular medication is for, and for clinicians to interpret the indication accurately.

The idea of having a patient-facing website or app was also attractive to most participants, so long as it was straightforward to access. Many participants reported they currently use lists to manage their medications; a patient website or app is similar, just in a digital format. Reducing friction in the sign-in process could help facilitate the adoption and uptake of a website or portal for patients’ use but would not accomplish all of the goals of having RFU written on medication bottles, especially to allow others to determine RFU.

Extensive work has been conducted in the area of RFU improving safety, like the work of Bosch-Lenders recommending the addition of RFU on medication labels to support older adults safely using medications and to improve patient adherence [6, 29]. The latter study by Garada, Schiff, and colleagues involved researchers interviewing patients regarding the use of putting RFU on medication labels indications as well. Additional work by Schiff and colleagues has examined the addition of medication indications via “indications based” prescribing to allow for easier and faster addition of medication indications, to enhance medication safety, and to improve clinician communication [6, 30, 31]. This paper builds on this existing work by highlighting some additional benefits to adding RFU on medication labels, such as keeping track of medication despite changes in brand or packaging and the value in having RFU on medications in emergency situations. Additionally, the patients interviewed described using RFU to support decision making regarding their medications, as opposed to serving as a reminder to take a medication.

Ensuring the addition of RFU as a matter of course on prescriptions and medication labels will require collaboration by a number of stakeholders before it becomes commonplace. This may take the form of engaging with regulatory bodies, interest groups, or individual prescribers for a more grassroots approach [32]. Additional research engaging patients from other healthcare models and geographic locations, as well as research quantitatively investigating improvements to clinical practice as a result of sharing RFU, would strengthen the case for including RFU on prescriptions and medication labels.

### Limitations

While the 20 participants were varied in gender, age, and understanding of their medications, they were all from a single geographic region in a publicly funded healthcare system. The findings may be different from other participants or in other areas. Currently, patients in this region lack electronic access to their own healthcare records (i.e., via a patient portal). These patients may thus lack an understanding of how these systems could benefit them; however, the role that communicating RFU in could play benefiting them is universal. Additionally, patients volunteered to participate in this study, leading to a potentially biased sample who may have a stronger understanding of their medications than the general population. This study did not capture the experiences of people taking more than ten medications or those with living with dementia. Information on the patients’ mental health history was also not collected. Despite these limitations, the patients interviewed provided insight as to how RFU may assist with medication use to those providing care to others.

## Conclusion

This study demonstrated the value of RFU for patients and explores a number of ways it could be effectively communicated, with respect to both format and delivery method. These results can be used to advocate for patients to have access to RFU on their medication labels, to help patients make decisions about taking their medications, and could improve patient adherence with prescribed medications. By keeping in mind the diverse group of people who may ultimately need to learn a medications’ RFU from its label, prescribers and pharmacists should ensure that the RFU information included is understandable by a wide range of people.

## Supplementary information

**Additional file 1.**

## Data Availability

The data that support the findings of this study are available on request from the corresponding author C.W. The data are not publicly available due to the data containing information that could compromise participant privacy.

## Abbreviations

RFU: Reason for use
SRQR: Standards for Reporting Qualitative Research
STI: Sexually transmitted infection
PHI: Protected health information
