# Supplementary material for: How do I keep myself safe? Patient perspectives on including reason for use information on prescriptions and medication labels: a qualitative thematic analysis
Source: J Pharm Policy Pract. 2020 Oct 7;13:63. doi: 10.1186/s40545-020-00268-6 (PMC7541269; doi:10.1186/s40545-020-00268-6)
Supplement: Supplementary file 1 — Additional file 1. [file 40545_2020_268_MOESM1_ESM.docx]

**INTERVIEW QUESTIONS FOR PATIENTS**

*First, we are going to ask you a few questions about yourself.*

Demographic questions:

1. What is your age?
2. What is your gender?
   1. Man
   2. Woman
   3. Non-binary

*Questions related to your medication history*

1. How many medications do you take regularly?
2. In the last month, can you tell me what medications you took and why you took each of your medications?
3. Do you keep any lists of these medications (in a file, on a computer, etc.)?
4. Would you be comfortable with us taking a picture of these records? We will ensure that all identifying information is kept confidential and protected.

*We are going to talk about how you interact with the current system. In particular we are interested in something called “reason for use”. This is the reason a medication was prescribed to you. For example, the reason for use with Tylenol may be “a headache” our “arthritis pain”.*

Questions related to how you function in the current system:

1. How do you currently find out what your medications are for?
2. Where do you place this information? (Potential probes: Do you write it down? Do you keep it put in your phone? Do you make medication lists?)
3. In the past, has the reason for use information been shared with you?
   1. Who shared this type of info with you? Pharmacist? Physician?
   2. When did they share it? Did you have to ask to find out or was this info provided?
   3. Please provide an example of what type of language was used to explain a medication. How useful was this?
   4. Was there a time that the reason for using a medication a professional provided was not clear or useful? Please explain.
   5. Can you recall a time that learning the reason for using a medication was particularly helpful for you? Please tell me more.
4. Who else is aware of the reasons you are taking your medications?
5. Can you recall a time when you did not know the reason for a medication? What implications did this have?
   1. What did you do to find out (if appropriate to ask)? (ask user to draw out the steps they took to find out the reason for use information)
6. On a scale of 1 to 5, with 5 being “very important,” how important is it for you to know what your medication are for? Please tell us more.

*We are going to talk about what would happen if the "reason to use" was added to the computer systems that doctors, pharmacists, and nurses use to help you manage your medications.*

Questions related to being provided with “reason to use” information:

1. How would you feel if the reason for use was shared with your pharmacist on every prescription, including refills?
2. How should the reason for use information be presented to you? (Probing questions: Would you like it to be on your prescriptions? Receipts? Medication lists? In an app or website that you use to view or access your prescriptions?)
3. How would the reason for use information affect your ability to make decisions about your medications?
4. Who else should have access to your reason for use information? This can be anyone in your life such as healthcare professionals, care providers, or family members. Why?

**Prescription Label Design Workshop**

*Now we are going to design a prescription label that would hypothetically incorporate the “reason to use” information.* (Hand participant example prescription label template)

*Place an ‘X’ where you would want to see “reason to use” information.*

Follow-up Questions:

1. Why did you choose this location?
2. How much detail would you expect? (Potential probes: Short-hand? Long-hand?)
3. If you could rearrange the information on this prescription label, would you?
   1. If yes, please show us. (Hand participant blank prescription label template, corresponding prescription label information, and tape)
   2. Why did you choose the layout you did?
4. Do you have any concerns regarding the sharing of reason for use information with the pharmacist or on your prescription labels? If so, what?
5. On what occasion(s) would you like this information on your prescription label?
6. On what occasion(s) would you not like this information on your prescription label?
   1. (if they acknowledge privacy concern) How you suggest affording privacy concerns?
7. Do you prefer the phrase ‘reason to use’ or ‘reason for use’?
8. Who do you think would benefit from this new design?
9. How will you use this new design?
10. Does this adding reason to prescription labels solve the problem we’re trying to solve?
11. How might we think about this design differently?
12. Is there any other way to accomplish this design problem?
13. What do you believe is the end result of doing this?
14. Do you have any final thoughts, comments, or concerns?
15. On a scale of one to 5, how much do you depend on your prescription label for proper medication management and/or knowledge?
